# Torsional Characteristics of Injection-Molded Hinges from Plastics and Glass Fiber-Reinforced Plastics

**DOI:** 10.3390/polym17192682

**Published:** 2025-10-03

**Authors:** Tran Minh The Uyen, Van-Thuc Nguyen, Xuan-Tien Vo, Pham Son Minh, Hai Nguyen Le Dang

**Affiliations:** Faculty of Mechanical Engineering, Ho Chi Minh City University of Technology and Education, Ho Chi Minh City 71307, Vietnam; uyentmt@hcmute.edu.vn (T.M.T.U.); nvthuc@hcmute.edu.vn (V.-T.N.)

**Keywords:** ABS, torsional moment, glass fibers, pure polymer

## Abstract

This study investigates the torsion characteristics of injection-molded flexural hinges manufactured from common polymers and plastic-based composites. The compliant mechanism provides a nearly constant torque over a specific rotational period. The flexural hinges are created via the injection molding technique, which has the advantage of mass production and low price. The injection plastics are pure polypropylene (PP), acrylonitrile butadiene styrene (ABS), and polyamide 6 (PA6), and the injection composites are PA6 combined with glass fibers. The torsional moment of the ABS flexural hinge ranges from −0.2 to 0.94 N∙m. The torsional moment of the PP polymer typically ranges from −0.6 to 0.8 N∙m. The torsional moment of the PA6 polymer ranges from −0.2 to 1.0 N∙m. Interestingly, the torsional moment diagram for this polymer is comparable to that of ABS, with a stable pattern in both positive and negative ranges. Furthermore, in other words, the PP flexural range is greater than the ABS range. Both ABS and PA6 flexural hinges have a higher level of stability compared to the PP one due to the higher elastic modulus and higher strength of these polymers than the PP polymer. The PP flexural hinge has the lowest negative torsional moment (−0.6 N∙m) compared to ABS and PA hinges. PA6 flexural hinges also have the most stable torsional moment compared to pure polymer varieties. Adding 5% to 10% fiberglass (FG) significantly improves the torsional moment of composite flexural hinges. More flexural hinges from different polymer types should be investigated. Further research should conduct some statistical analysis to clarify the variations between the torques for the various materials. The findings improve our understanding of plastic flexure hinges and expand their applicability.

## 1. Introduction

A flexible hinge is a mechanism that allows the transmission or transformation of force and moment motion through elastic deformation of the structure [1,2,3]. The movement of the flexible hinge causes its links to deform and store elastic energy. This energy is then released to help the mechanism perform a predetermined function [4,5,6]. Many types of flexible hinges can be classified based on geometric structure, kinematics, and compliant mechanisms, including bridge type, leaf spring, notch hinge, rotational hinge, bistable mechanism, and compliant mechanism [7,8,9,10].

Bistable and compliant mechanisms are mechanisms that have three equilibrium positions without using additional energy to maintain them in those states [11,12,13,14]. Unlike torsion springs, whose torque increases as rotation increases, the compliant mechanism provides a nearly constant torque over a specific rotational period [15,16,17]. Instead of using sensor control, the compliant mechanism passively maintains a constant torque. Potential applications include static and dynamic balancing of machinery, human joint rehabilitation devices, and human mobility aids.

Flexural type and material selections have a significant impact on flexural hinge performance. Flexural hinges can be made from metallic materials such as aluminum, titanium, copper, and shape memory alloys [18,19,20]. They frequently exhibit high mechanical properties and environmental stability. Metallic flexural hinges can be machined using traditional machining procedures or 3D printing, which is quite expensive. For example, Li et al. [21] developed an in-space optical camera to refine the design of a compliant focusing mechanism. To achieve the lowest possible weight, the compliant mechanism is made of 7075-T651 aluminum alloys. The optimization method’s focusing mechanism reduced the overall weight considerably and allowed the focal plane assembly to move within a 2 mm range. Pham et al. [22] designed a new two-degrees of freedom (DOF) high-precision compliant positioning mechanism created through an optimization process that incorporated Six Sigma analysis, the response surface method, and the finite element method. This compliant positioning mechanism consists of a right-circular hinge, leaf spring, and hollow flexure hinge. The optimal design was developed to assess its performance in a vibration-assisted milling experiment concerning surface roughness criteria. The outcomes show a good improvement in both the design approach criterion and the manufacturing criterion of surface quality. Triangular bi-axial flexure hinges’ flexibility is analyzed using titanium alloy TC4 by Lin et al. [23]. Following that, the analysis model is verified using both linear and nonlinear finite element methods. The results show that the multi-island genetic algorithm successfully optimized the hinge’s physical dimensions and improved its performance. Interestingly, Mozhi et al. [24] created a shape memory alloy (SMA)-actuated monolithic compliant gripping mechanism that can be used for grasping tasks appropriate for microassembly and micromanipulation. The report shows that the gripper’s displacement amplification gain of 3.7 enables a maximum tip displacement of 1.2 cm, giving it a geometric advantage and good handling range that traditional grippers are unable to achieve. However, the material and machining costs are expensive, as they are created by single-piece flow, limiting their applications. The machining process that could create a complex and precise shape of the flexural hinge usually requires enormous effort on high-end processes such as metallic 3D printing, computer numerical control (CNC) machining, and electrical discharge machining (EDM). Therefore, finding other materials such as polymers and composites could lower costs and increase the appearance of flexural hinges in the industrial field.

Applying injection molding for flexural hinges could dramatically reduce the manufacturing cost. Injection molding possesses the advantages of industrial availability, high accuracy, and low cost. Injection molding is a widely used technique that can create plastic components at a mass production level [25,26]. In addition, many polymers and composites can be formed by injection molding, for instance, polypropylene (PP), polyvinyl acetate (PVA), polyamide 6 (PA6), acrylonitrile butadiene styrene (ABS), polyethylene (PE), and their polymer-based composites reinforced by glass fiber or carbon fiber. Minh et al. [27] investigated the amplification of compliant hinges with bridge-type shapes by using an injection molding process with many plastic types. The injection-plastic hinges could be manufactured in large quantities at a low cost and with high productivity. The amplification ratio values for the high-density polyethylene (HDPE), PP, and ABS flexure hinges are 2.83–5.4, 4.1–6.73, and 4.81–8.81, respectively. Overall, the ABS flexure hinge could be superior to the PP flexure hinge in terms of amplification ratios. HDPE flexure hinges have the lowest amplification ratio among all these plastic types. However, the investigations about the injection flexural hinge are still rare and need further investigation.

This study tries to explore the torsion characteristics of injection-molded flexural hinges from some common plastics and plastic-based composites. The injection plastics are pure PP, ABS, and PA6, and the injection composites are PA6 mixed with glass fibers. They are widely used plastics and composites with low prices and availability. Therefore, this study selected them for testing, as the results can be easily applied. After injection, the flexural hinges are tested via the torsion test. Furthermore, a comparison of these injection-molded plastic-based flexural hinges is undertaken to determine the best selection. The discoveries improve our understanding of plastic and composite flexure hinges and broaden their applications.

## 2. Experimental Methods

**Figure 1:**
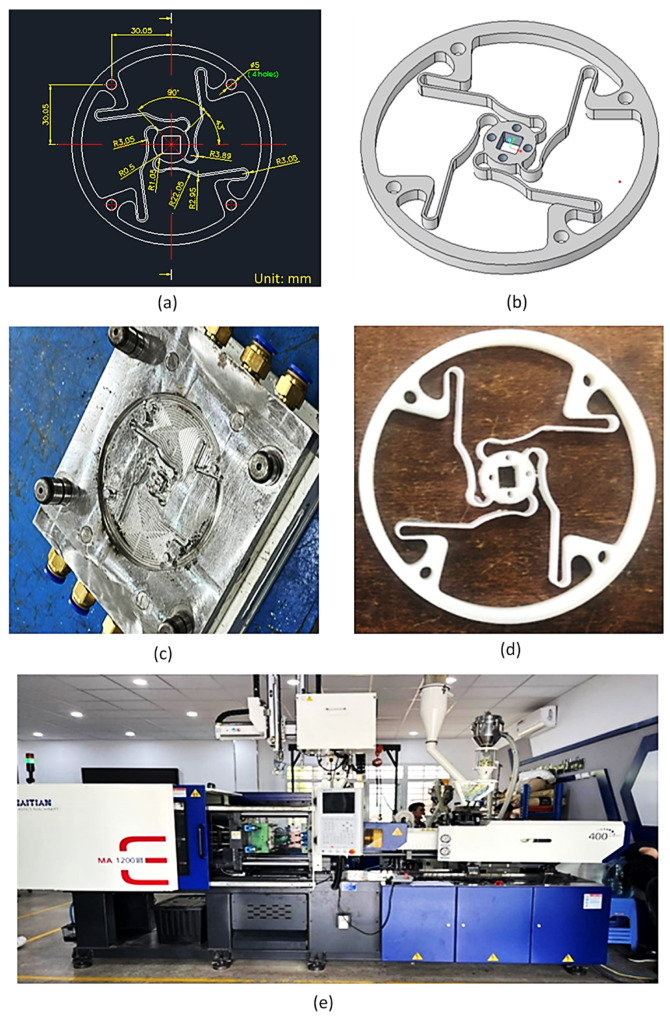
Flexure hinge design and injection-molded samples: (**a**) 2D design, (**b**) 3D design, (**c**) injection mold, (**d**) injection-molded hinge, and (**e**) MA 1200III (Haitian, China) injection molding machines.

The model is designed, then the injection mold is manufactured for this model, and the mold is processed and assembled. Figure 1 shows the design of the compliant hinge, the mold, the injection-molded hinges, and the injection molding machine. The mechanism has a circular shape with an outer diameter of ∅100 mm and a contour thickness of 5 mm. This model has a total of 4 wires with a thickness of 1 mm connected from the circular cylinder in the center to the circular contour on the outside. These four wires can be extended depending on the twist angle. The geometric dimensions of the tested joints are determined based on the injection ability of the injection molding machine. Then the model is injection molded with ABS, PP, PA6, and PA6 with glass fiber contents ranging from 5% to 30%. The injection molding machine type MA 1200III (Haitian company, Ningbo, Zhejiang, China) is used to create the injection molding flexural hinge. ABS 750 SW polymer is manufactured by Kumho Petrochemical, Korea. PP polymer named Advanced-PP 1100 N is supplied by Advanced Petrochemical Company, Saudi Arabia. PA6 and PA6 30% fiberglass (FG) are provided by the Envalior company, AT Emmen city, Netherlands.

In injection molding, many parameters need to be set that affect the physical and chemical properties of the product. This study considers many critical injection parameters, including filling pressure, filling speed, filling time, packing pressure, packing time, and temperature. Injection pressure is the value of force applied by the injection molding machine through the screw or plunger at a specific velocity to fill the cavity to 95–98% by volume. Packing pressure occurs in the molten plastic when it has been filled to the desired volume. Its goal is to “pack” additional material into the mold to compensate for shrinkage when the product cools. Before molding the product, the plastic particles are dried at 80 °C for ABS and PP plastic and 110 °C for PA6 plastic for 2 h. Initially, some basic injection parameters are examined to ensure a good injection shape and avoid some basic molding defects such as short shots, burn marks, and warping. After that, the experimental parameters are set up, as shown in Table 1. With the presence of additional glass fibers, the filling pressure and packing pressure are increased compared to the pure PA6 case to ensure good filling conditions. The material quality, injection molding quality, process parameters, and dimensional check are controlled to ensure the repeatability of the injection molding process. Each test condition number is examined via three samples.

**Figure 2:**
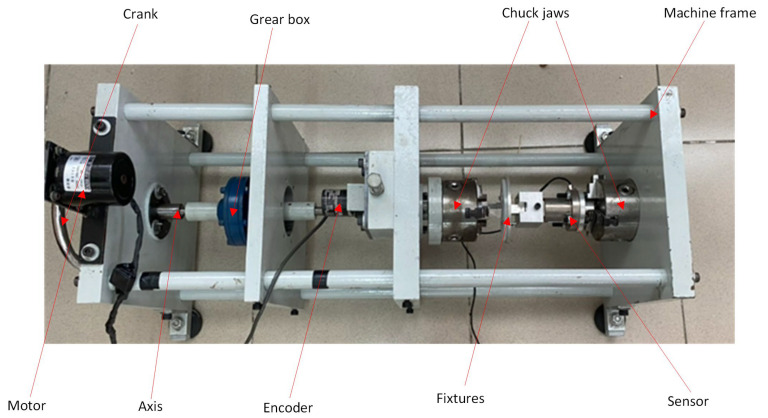
Torsion test machine for plastics and composite flexural hinges.

Figure 2 shows the torsion test machine for plastic and composite flexural hinges. The structure of the torsion testing machine includes the machine frame, which plays an important role in the torsion testing machine, which is manufactured based on ASTM A938, Standard Test Method for Torsion Testing of Wire [28]. The machine frame helps to withstand and provide capacity to support other parts in the torsion testing machine system, helping to position other parts in the torsion testing machine, such as the reducer, chuck, adapter, and control devices. It helps to connect and maintain the stability of these parts, ensuring the efficient operation of the torsion testing machine. The torsion angle is −40–100°, repeating at a cycle range of 200. The torsional diagram is separated into the negative and positive lines, with 100 cycles for each line. For each injection material type, five hinges are injection molded for an individual measurement. The average results of five samples are calculated and shown in this report.

The torsion test equipment used a 40 W AC synchronous motor to apply a controlled load of 0–300 Nm, with torque transmitted via a 1:2 timing belt pulley. The motor operated at 10 RPM, ensuring stable moments with minimum slippage. A hand wheel allowed manual shaft rotation. The torque was amplified using a 1:50 planetary gearbox. Rotation angles were measured using a belt tension mechanism and a 1000-pulse encoder with a belt drive ratio of 1:1. The data-collecting unit features three-jaw chucks for accurate specimen alignment and a torque sensor with an error margin of ±0.005 N·m for real-time data capture. The system’s inputs are the motor’s rotation and torque. The measured torque and rotating angle are returned as output. Next, the machine’s kinematic balance equation is presented as follows:

One motor rotation × 1/2 × 1/40 = Angle. (1)

With this motor running at 10 rpm, the encoder’s recorded angle is 45 degrees per minute, which matches the machine’s twist rate. The machine’s power is lost through the bearings, toothed belts, couplings, and gearbox. To compute the angle of twist (θ) for a linear elastic material, apply the following equation:

(2) $$\theta =\frac{TL}{JG}$$

where T is the applied torque (in N.m). J is the second polar moment of the cross-sectional area, which is determined by the test specimen’s cross-section. The following are two common cross-sections. G is the modulus of stiffness or shear modulus (Pa), and it can be determined using Equation (3):

(3) $$G=\frac{TL}{\theta J}$$

This equation shows the direct proportional relationship between torque (T), length (L), angle of twist (∅), inverse ratio of polar moment of the area (J), and modulus of stiffness (G).

## 3. Results and Discussion

### 3.1. Polymers Flexure Hinge

This section discusses how different types of pure polymers affect the torsional moment. The original torsional moment, its division into positive and negative diagrams, and some comparisons are discussed to help illustrate the torsional properties of the injection-molded flexural hinges.

**Figure 3:**
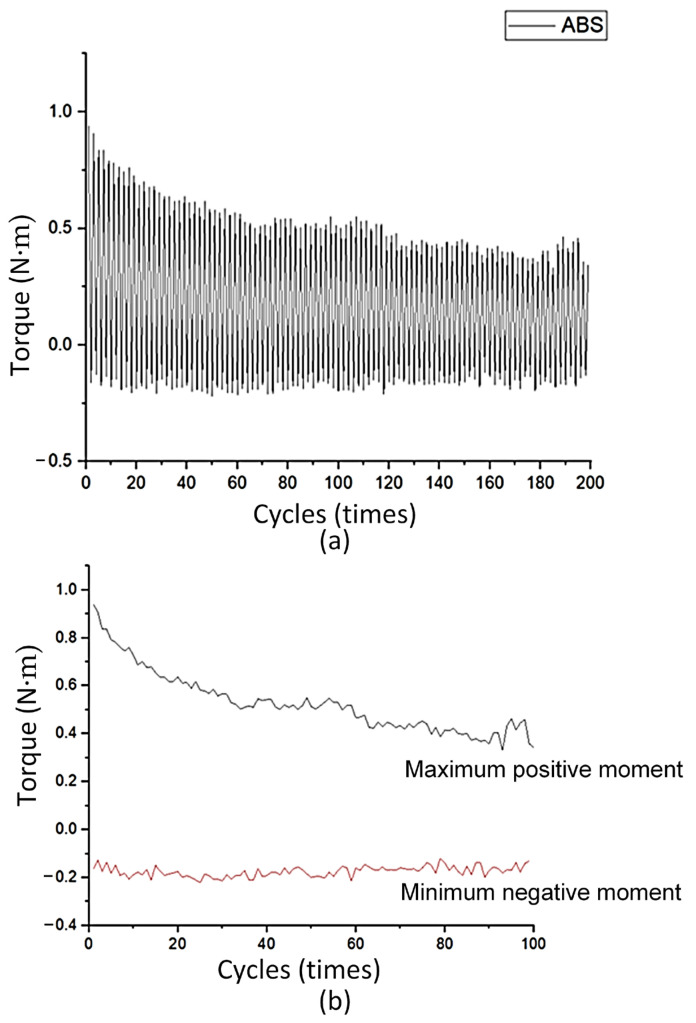
Torsional moment of the ABS flexural hinge: (**a**) torsional moment diagram of the ABS flexural hinge, and (**b**) maximum positive and minimum negative moment diagrams.

Figure 3 shows the torsional moment diagram of the ABS flexural hinge. The torsional moment of this polymer varies from −0.2 N∙m to 0.94 N∙m, as shown in Figure 3a. Figure 3b provides more details about the minimum negative and maximum positive moments separately. The negative line mostly varies about −0.2 N∙m, indicating the stable performance of the ABS flexural hinge at this range. On the contrary, with the positive line, the torsional moment suffers a steady decline. The reason is the polymer chain rearrangement at strain under many deformation cycles, leading to the gradual reduction in the positive range [29]. In addition, this difference in the positive and negative ranges could be the set range of the test and the design of the flexural hinge. The negative angle is set at −40°; therefore, its absolute value is 40°, which is greatly lower than the positive angle of 100°. Another reason is that the hinge shape is not symmetrically designed.

**Figure 4:**
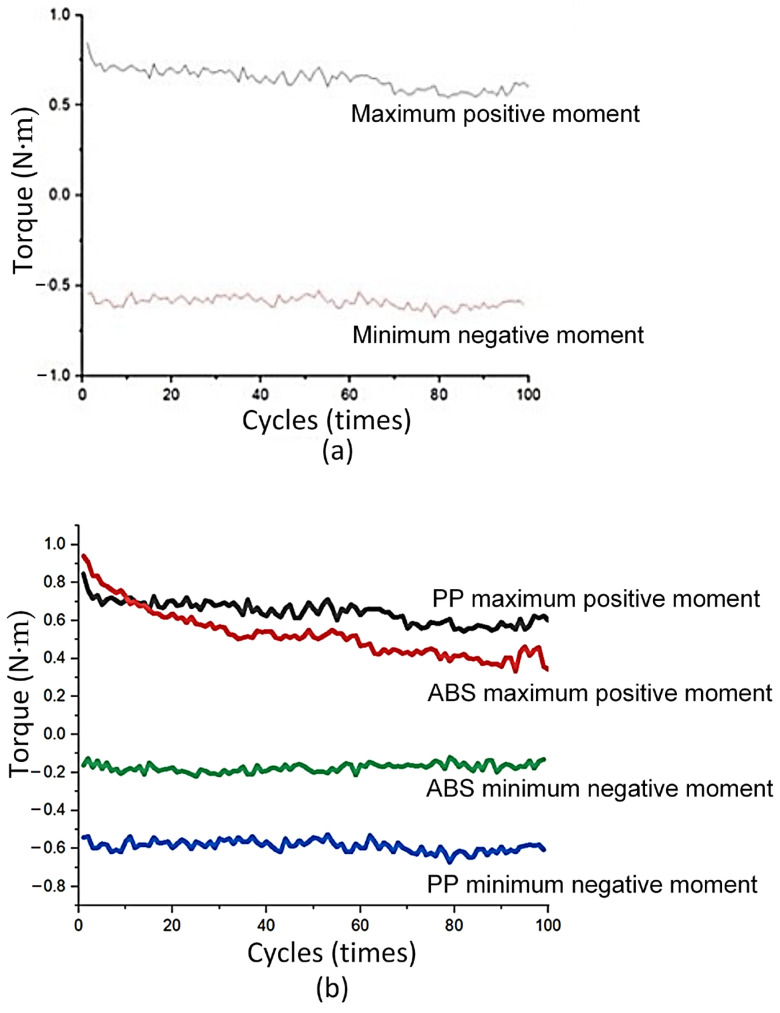
Torsional moment of PP flexural hinge: (**a**) positive and negative momentum diagrams of PP flexural hinge, and (**b**) positive and negative momentum diagrams of ABS and PP comparison.

Figure 4 shows the torsional moment diagrams of the PP flexural hinge. The torsional moment of this polymer mostly oscillates from −0.6 N∙m to 0.8 N∙m. Compared to the ABS flexural plastic hinges, the absolute value of the negative torsional moment of the PP flexural hinge is lower, indicating a stronger resistance to plastic deformation. The negative range also reveals a stable torsional moment value around −0.6 N∙m. On the contrary, at the positive torsion range, the PP flexural plastic hinge performs a lower maximum torsional moment than the ABS one (0.8 N∙m and 0.94 N∙m). Despite having a lower maximum torsional moment, the PP flexural hinges have a lower decreasing speed over 100 cycles, reaching the lowest point at about 0.5 N∙m, as shown in Figure 4a. In reverse, the ABS one has a faster decreasing speed, having a lowest point of 0.4 N∙m, as shown in Figure 4b. In other words, the PP flexural hinge has a more stable performance than the ABS one and a higher value in negative torsional moment.

**Figure 5:**
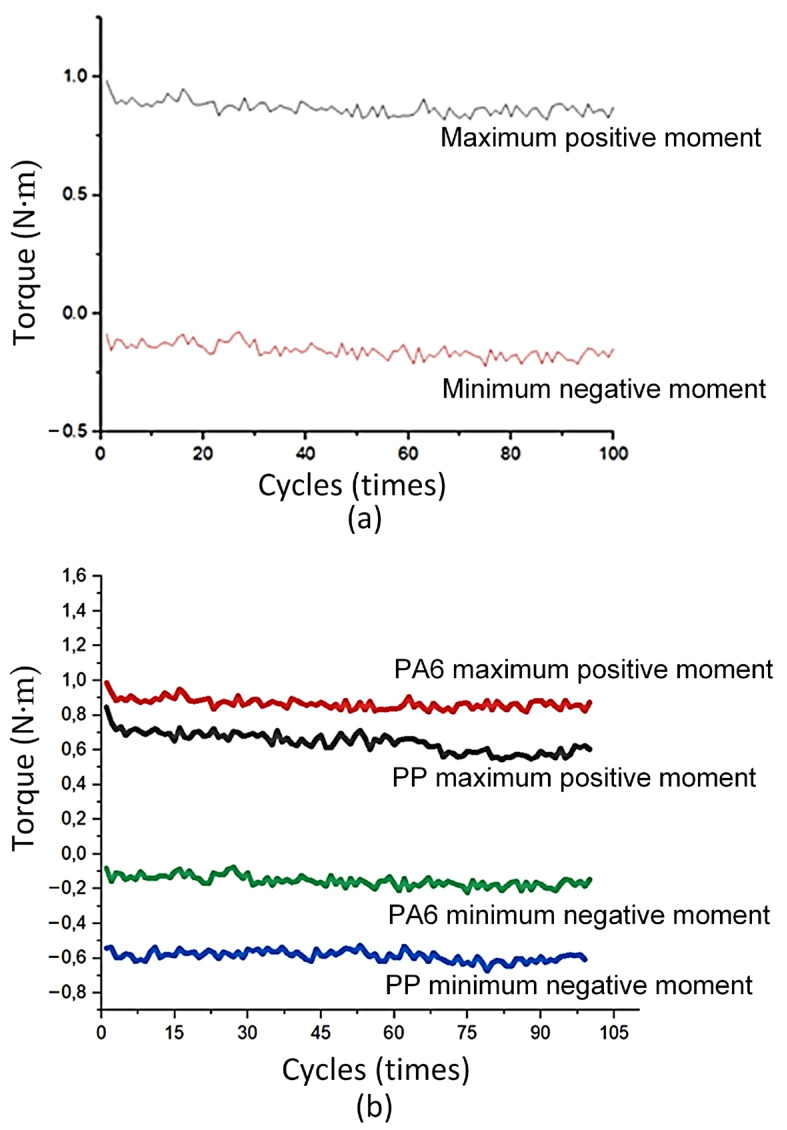
Torsional moment of PA6 flexural hinge: (**a**) positive and negative momentum diagram, and (**b**) PA6 and PP comparison.

Figure 5 shows the torsional moment diagram of the PA6 flexural hinge. The torsional moment of this PA6 polymer mainly varies from −0.2 N∙m to 1.0 N∙m, as shown in Figure 5a. Interestingly, the torsional moment diagram of this polymer is similar to the ABS one, having a stable pattern in both positive and negative ranges. Additionally, the PA6 flexural hinges have a higher value at the positive range than the ABS range (1.0 N∙m compared to 0.8 N∙m). In the negative range, the PA6 has the higher torsional moment when considering the absolute value (0.6 N∙m compared to 0.2 N∙m), as shown in Figure 5b. In addition, both ABS and PA6 flexural hinges have a higher level of stability compared to the PP one. The reason could be the higher elastic modulus and higher strength of these ABS and PA6 polymers than PP [30]. The stronger materials could perform better in torsional moments [31].

### 3.2. Composite Flexure Hinges

In this section, the PA6-based composite is examined with the glass fiber content varying from 5% to 30%. Firstly, the torsional moment of PA6 with 5% FG is investigated, comparing it to the pure PA6 case. Thereafter, the other percentages, such as 10%, 15%, 20%, 25%, and 30% FG, are surveyed.

**Figure 6:**
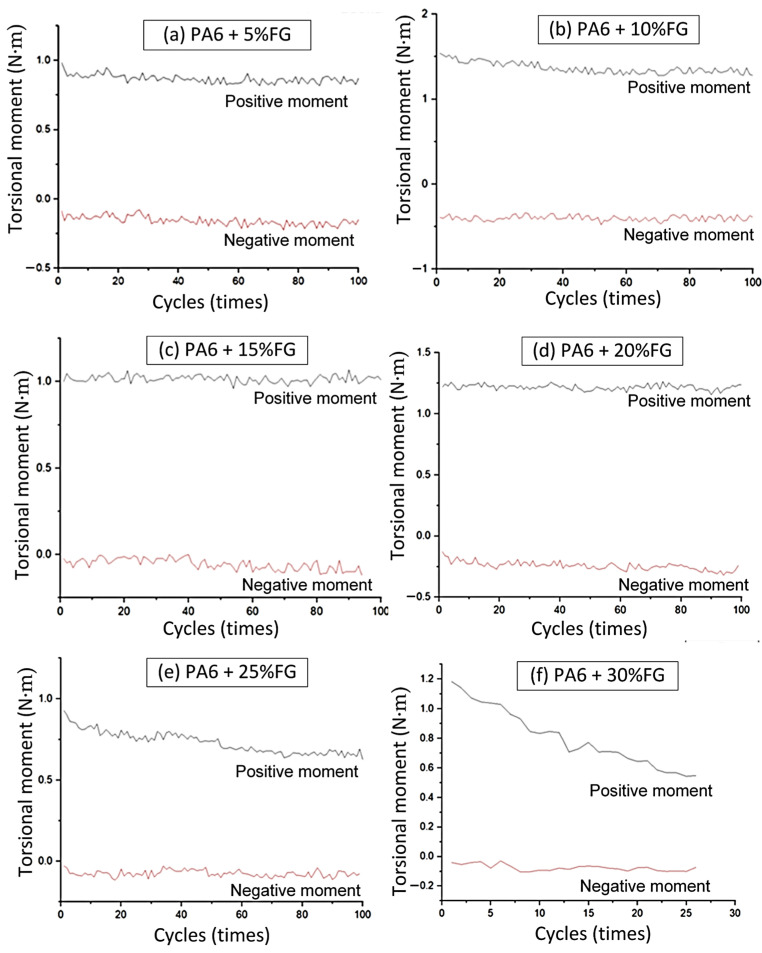
Torsional moment of PA6-based composite flexural hinges: (**a**) PA6 5% FG, (**b**) PA6 10% FG, (**c**) PA6 15%FG, (**d**) PA6 20% FG, (e) PA6 25% FG, and (**f**) PA6 30% FG.

Figure 6 shows the torsional moment diagram of PA6 mixing with a 10–30% FG flexural hinge. In general, considering all pure PA6 and PA6 + 5–30% FG composite flexural hinges, the longest range of torsional moment is −0.5–1.5 N∙m, as shown in Table 2. Glass fibers could increase the strength and elastic modulus of the PA6 composite; however, the ductility could suffer a sudden reduction when adding too many glass fibers [32]. This could be the reason for the lower torsional moment range of the high FG content compared to the lower one. The higher glass fiber content could further increase the strength of the composite flexural hinges, therefore improving the torsional moment of the flexural hinge. Adding glass fibers could reinforce the PA6 matrix; however, the torsional moment does not simply improve when the glass fiber percentage increases. From 5% to 10% FG, adding glass fibers to the PA6 matrix mostly helps improve the torsional moment of the composite flexural hinges. Moreover, both the positive and negative torsional moment values also experience a stable condition when testing at all cycle ranges. This improvement comes from the suitable reinforcement of the matrix, while the ductility of the materials is still good. This 5% to 10% FG composite is the optimal range, resulting in a good torsional moment. However, further adding 15% to 30% FG, the torsional moment of the PA6-based composite flexural hinges mostly fluctuates around −0.1 to 1.0 N∙m, pointing out the limitation of the reinforcing effect. Compared to the other materials, the composite thin-walled hinge created from carbon fiber could achieve a higher torsional moment of 18.48 N∙m; however, the angle range was limited to only 10–15.4° [33]. Moreover, carbon fiber also has stronger tensile strength and elastic modulus; therefore, it could perform better in terms of torsional moment values. Additionally, plastic flexural hinges are cheaper than the metallic ones, as shown in Fava et al.’s study [34].

In general, among the three pure polymers, the PP flexural hinge could achieve the lowest negative torsional moment of −0.6 N∙m compared to ABS and PA flexural hinges. In reverse, the PA6 could gain the highest positive torsional moment of 1.0 N∙m. PA6 flexural hinges also achieve the most stable torsional moment compared to the other pure polymer types. In addition, considering the PA6-based composite flexural hinge, from 5 to 20% FG, the torsional moment also presents a stable torsional moment range. In addition, adding glass fibers from 0% to 10% helps increase the performance of the flexural hinges, as both the positive and negative moments experience a great improvement. Remarkably, at 5% FG, the composite flexural hinges gain their best torsional moment range.

## 4. Conclusions

This study investigates the torsion properties of injection-molded flexural hinges made from common polymers and plastic-based composites. The injection plastics are pure PP, ABS, and PA6, whereas the injection composites are PA6 combined with glass fibers. Torsion testing is used to evaluate the flexural hinges following injection. The discoveries advance our understanding of plastic flexure hinges and broaden their applications. Notable remarks that may be highlighted are as follows:

- The torsional moment of the ABS flexural hinge varies from −0.2 to 0.94 N∙m. The negative line mostly varies about −0.2 N∙m, indicating the stable performance of the ABS flexural hinge at this range. On the contrary, with the positive line, the torsional moment suffers a steady decline.
- The torsional moment of the PP polymer mostly oscillates from −0.6 to 0.8 N∙m. Compared to the ABS flexural plastic hinges, the absolute value of the negative torsional moment of the PP flexural hinge is lower, indicating a stronger resistance to plastic deformation. On the contrary, at the positive torsion range, the PP flexural plastic hinge performs a lower maximum torsional moment than the ABS one. Moreover, the PP flexural hinges have a lower decreasing speed over 100 cycles, reaching the lowest point at about 0.5 N∙m. In other words, the PP flexural hinge has a more stable performance than the ABS one and a higher value in negative torsional moment.
- The torsional moment of the PA6 polymer mainly varies from −0.2 to 1.0 N∙m. Interestingly, the torsional moment diagram of this polymer is similar to the ABS one, having a stable pattern in both positive and negative ranges. Additionally, the PA6 flexural hinges perform at a higher value in the positive range than the ABS range. In the negative range, the PA6 has the highest torsional moment when considering the absolute value. Both ABS and PA6 flexural hinges have a higher level of stability compared to the PP one due to the higher elastic modulus and higher strength of these polymers than the PP polymer.
- Among three pure polymers, the PP flexural hinge could achieve the lowest negative torsional moment of −0.6 N∙m compared to ABS and PA flexural hinges. In reverse, the PA6 could gain the highest positive torsional moment of 1.0 N∙m. PA6 flexural hinges achieve the most stable torsional moment compared to the other pure polymer types, indicating their advantages.
- Adding from 5% to 10% FG mostly helps improve the torsional moment of the composite flexural hinges. Moreover, both the positive and negative torsional moment values also experience a stable condition when testing at all cycle ranges. This improvement comes from the suitable reinforcement of the matrix, while the ductility of the materials is still good. However, further adding 15% to 30% FG, the torsional moment mostly fluctuates around −0.1 to 1.0 N∙m, pointing out the limitation of the reinforcement effect. PA6 mixed with 10% FG appears to be the optimal material among the surveyed range. The results broaden the application of injection-molded plastic flexure hinges and enhance our understanding of them. Further investigation should conduct some statistical analysis to clarify the differences between the torques for the different materials.

## Figures and Tables

**Table 1:** Injection parameters for pure PP, ABS, and PA6 polymers and PA6-based composite flexural hinges.

Materials	Filling Press. (MPa)	Filling Speed Rate (g/s)	Filling Time (s)	Packing Press. (MPa)	Packing Time (s)	Temp. (°C)
Pure PP	5.5	138.6	2	5.1	1	210
Pure ABS	9.5	138.6	2	8.7	1	200
Pure PA6	10.0	138.6	2	8.8	1	270
PA6 + 5% GF	10.0	138.6	2	8.8	1	270
PA6 + 10% GF	10.0	138.6	2	8.8	1	270
PA6 + 15% GF	10.5	138.6	2	9.3	1	270
PA6 + 20% GF	10.5	138.6	2	9.3	1	270
PA6 + 25% GF	10.5	138.6	2	9.3	1	270
PA6 + 30% GF	10.5	138.6	2	9.3	1	270

**Table 2:** Comparisons of torsional moment between pure polymer and composite flexural hinges with other compliant flexural hinges.

Materials	Torsional Moment (N∙m)	Characteristics of the Torsional Moment Diagrams
ABS	−0.2 to 0.94	Non-stable positive range with fast decreasing
PP	−0.6 to 0.8	Quite stable positive range, best negative value
PA6	−0.2 to 1.0	Stable positive range
PA6 + 5% FG	−0.5 to 1.0	Stable positive range, higher positive value than pure PA6
PA6 + 10% FG	−0.5 to 1.5	Stable positive range, highest positive value among all PA6-based composites.
PA6 + 15% FG	−0.1 to 1.0	Stable positive range, low negative value
PA6 + 20% FG	−0.25 to 1.25	Stable positive range, low negative value
PA6 + 25% FG	−0.1 to 0.75	Quite stable positive range, low positive value
PA6 + 30% FG	−0.1 to 1.2	Non-stable positive range with fast decreasing

## Data Availability

The data used to support the findings of this study are available from the corresponding author upon request.
